# The effects of the aeroball on plantar pressure during isometric hip contractions

**DOI:** 10.1186/1757-1146-7-S1-A103

**Published:** 2014-04-08

**Authors:** Kyungock Yi, Haelee Moon, Namjeong Son, Jaewon Choi, Haeryoung Won, Kyungsun Kim, Chanmi Kim, Jihee Yi, Hwalee Kim

**Affiliations:** 1 Division of Human Movement Studies, College of Health Science, Ewha Womans University

## 

Many female college students in South Corea suffer from postural problems and deformities in their lower extremities. In particular, many females exhibit knee valgus or varus. These problems can arise from a variety of causes, from the use of elevated heels, to years of carrying heavy backpacks, and muscle imbalance. There are many different types of exercises to address these problems, but isometric hip contractions are a simple movement that can be done anywhere at any time. This study tested whether foot pressure variables of isometric hip contractions could be improved through the use of an aeroball in order to correct knee deformities.

The purpose of this study was to evaluate the effects of the aeroball on plantar pressure during isometric hip contractions. Subjects for this study were 39 female college students. Subjects' plantar pressure was gauged with a Zebris (Germany) pressure plate while they performed 30 second isometric hip contractions with and without an aeroball (maker, diameter). Independent variables for the study were isometric hip contractions with and without the aeroball. Dependent variables were plantar pressure variables, such as length of x and y axis, path area, path length, and average velocity. All dependent variables were significantly higher with the aeroball.

The increased length of the x and y axis, and the larger path area, and path length all demonstrate greater movement during contractions. Thus, with the aeroball, subjects pushed their hips forward more with greater exterior rotation at the thigh, resulting in greater movement of pressure at the feet. In addition, the higher velocity for the aeroball demonstrates that subjects contracted their muscles more forcefully during the 30-second contraction interval.

In conclusion, pressure plate analysis revealed that isometric hip contractions were more effective with an aeroball. Future studies will build upon these results to evaluate the corrective effects of isometric hip contractions, especially on knee varus and valgus. Furthermore additional studies will investigate the relationship between diminished muscle function in the inner thighs, hips, and posterior chain, and postural problems.

**Table 1:** The differences in pressure variables according to aeroball usage

dependent variable	N	Mean(±SD)	mean diff.	t	P
wob_Length_of_x_axis	39	8.79(±2.28)	-1.49	-3.680	.001**
aeroball_Length_of_x_axis		10.27(±2.62)			

wobl_Length_of_y_axis	39	15.15(±6.90)	-2.46	-2.130	.040*
aeroball_Length_of_y_axis		17.61(±6.06)			

wob_Path area	39	111.77(±73.44)	-36.32	-2.619	.013*
aeroball_Path area		148.09(±77.24)			

wob_Path_length	39	297.01(±108.37)	-83.05	-3.804	.001**
aeroball_Path_length		380.07(±150.65)			

wob_Average_Velocity	39	10.09(±3.67)	-2.82	-3.803	.001**
aeroball_Average_Velocity		12.91(±5.12)			

*p<.05, **p<.01

wob; without ball
